# Distal interphalangeal joint arthrodesis with Herbert screw

**DOI:** 10.1186/1753-6561-9-S3-A46

**Published:** 2015-05-19

**Authors:** Shaher El-Hadidi

**Affiliations:** 1 Jordan University Hospital, Amman, 11942, Jordan

## Summary

Fifteen patients underwent arthrodesis of finger distal interphalangeal joint (DIP) and/or thumb interphalangeal joint (IP). Only Hebert screw intramedullary compression fixation was used. There was one faulty proximal thread placement with some pain, but the patient refused further surgery. Full union was achieved in 14 of the cases, with prominent hardware in one and intolerance to cold in two.

## Introduction

Conditions that cause pain, instability or joint deformity are common indications for distal interphalangeal (DIP) joint arthrodesis: There are many fixation techniques, which include: single Kirschner wire, crossed Kirschner wire, intraosseous wiring, bone peg, mini plate fixation, polypropylene peg, compression clamping, tension bands, Herbert screw and A-O compression screws. We found that the Herbert screw fixation was the most stable and the least complicated.

## Material and methods

Between 1994 and 1999, 15 DIP and/or thumb interphalangeal (IP) joint arthrodesis procedures with Herbert screw were performed. There were eight male and seven female patients with thumb IP joint fusion and 11 DIP joint fusions. The 13 who (86%) attended follow-up were of mean age 26 years (15-51 years) and a follow-up period ranging from three to five years. The indications for fusion were failed tendon surgery, O-A from different causes, and congenital causes.

## Operative technique

The DIP joint was approached dorsally through a transverse incision V-extension bilaterally (double Mercedes-Benz star incision) ^6^. This approach allows wide exposure of both sides of the joint and minimizes injury to the arterial and venous structures in the deep dermal layer. The technique also avoids injury to the germinal nail matrix.

The extensor tendon is transversely transected 1 to 2 mm proximal to the joint; the tendon is retracted distally and elevated slightly (approximately 1mm) from its insertion into the distal phalanx. Care is taken at this point to make this dissection subperiosteal to avoid injury to the nail germinal matrix.

The collateral ligaments are then released from the middle phalanx and the joint is hyperflexed giving wide joint exposure. Marginal osteophytes are removed with a synovectomy rongeur, the remaining cartilage and minimal subchondral bone is removed from each end of the joint with a small oscillating saw to allow opposing cancellous bone. Zero degree is the target in this fusion, as it is the most stable position there is no flexion, pronation or supination.

Herbert screw is used for fixation because of its smaller size compared to other screws. The No. 1 drill bit (a 2.4 mm drill bit) was not used in this operation; instead the No.2 drill bit (a 2.0 mm drill bit) was used to drill the distal phalanx in an antegrade manner placing the hole in the upper half of the distal phalanx, articular surface.

The same drill bit is then introduced from the fingertip down the whole shaft of the distal phalanx. The entry point in the middle phalanx is decided in the intercondylar sulcus in the upper half of the head of the middle phalanx and drilled down to its middle. The Herbert tap is introduced from the distal phalanx to the middle phalanx to the mid portion of the diaphysis to permit the leading threads of the Herbert screw to engage the endosteal cortex. The appropriate measured screw is selected and introduced while maintaining manual compression and the zero angle of arthrodesis. Care is taken at the end, to sink the head thread of the screw inside the tip of the ungula process.

## Results

**Figure 1:**
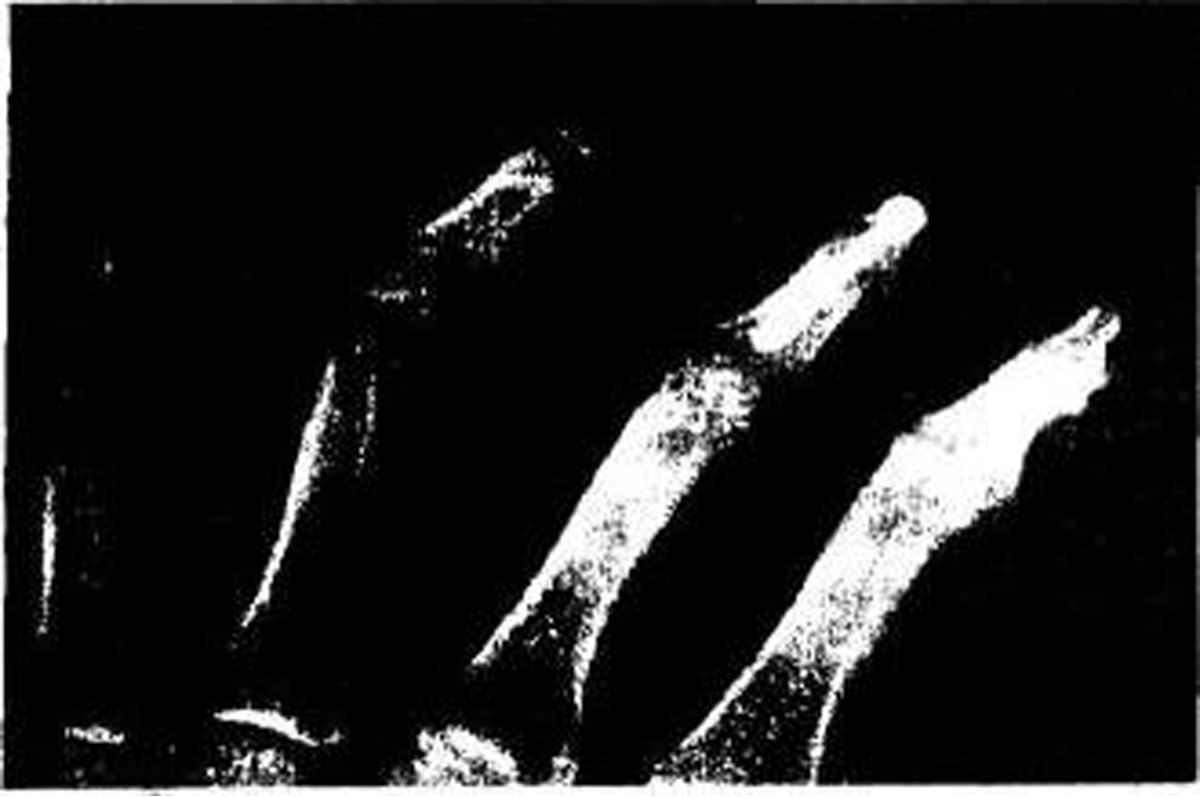
Full union.

Twelve out of 15 cases achieved full union (Fig. 1). Union was determined by radiographic trabecular bridging and clinical stability^2^. One case failed to unite at six months post operative two cases were lost to follow-up. The average time of the union was ten weeks (four to 20 weeks). Pain relief was apparent in all cases and improved with time. Prominence of hardware developed without shattering the terminal phalanx in one case in which the patient philosophically said “I learnt to live with it”!

## Discussion

Arthrodesis of small joints in the hand is indicated to relieve pain, restore stability and function and in some circumstances improve appearance. The goal is properly stated by Moberg and Henrickson: “The prime requisites of a good digital arthrodesis are painless and stable union in proper position and reasonable time”.

No single technique has gained 100% popularity for small joint arthrodesis. The principles for small joint arthrodesis are the same as for other joints, requiring bone-to-bone contact, adequate Reports in the literature indicate non-union rate between 0% and 30%. Faithful and Herbert reported 100% union rate in 11 DIP and two thumbs IP fusions. Stern and Fulton reported an 11% rate of non-union in three out of 37 DIP joint arthrodesis performed and our results are comparable to the reports in the literature.compression and immobilization.

The case that failed to unite was due to faulty proximal screw placement but the patient declined any further surgery. The incidence of cold intolerance in our patients is not different from that occurring after other types of fixation.

Stability and pain free DIP joint is well needed in bread winning age as in all of our patients for which the technique has been offered. When compared with other types of arthrodesis, it is superior in stability, design configuration, screw rigidity and ease of operative technique.

We conclude that Herbert screw is a feasible and adequate way of DIP joint arthrodesis.

